# Short-term Associations between Fine and Coarse Particulate Matter and Hospitalizations in Southern Europe: Results from the MED-PARTICLES Project

**DOI:** 10.1289/ehp.1206151

**Published:** 2013-06-18

**Authors:** Massimo Stafoggia, Evangelia Samoli, Ester Alessandrini, Ennio Cadum, Bart Ostro, Giovanna Berti, Annunziata Faustini, Benedicte Jacquemin, Cristina Linares, Mathilde Pascal, Giorgia Randi, Andrea Ranzi, Elisa Stivanello, Francesco Forastiere, the MED-PARTICLES Study Group

**Affiliations:** 1 Department of Epidemiology, Lazio Region Health Service, Rome, Italy; 2 Department of Hygiene, Epidemiology and Medical Statistics, Medical School, University of Athens, Athens, Greece; 3 Department of Epidemiology and Environmental Health, Regional Environmental Protection Agency, Piedmont, Italy; 4 Centre for Research in Environmental Epidemiology, Barcelona, Spain; 5 Air Pollution Epidemiology Section, Office of Environmental Health Hazard Assessment, California Environmental Protection Agency, Oakland, California, USA; 6 INSERM U1018, CESP–Centre for research in Epidemiology and Population Health, UMRS U1018, Respiratory and Environmental Epidemiology Team, University Paris Sud, Villejuif, France; 7 CIBER Epidemiología y Salud Pública (CIBERESP), Madrid, Spain; 8 Cancer and Environmental Epidemiology Unit, National Centre for Epidemiology, Carlos III Institute of Health, Madrid, Spain; 9 Unité Air Eau et Climat, Département Santé Environnement Institut de Veille Sanitaire, Saint-Maurice Cedex, France; 10 Epidemiology Unit, Local Health Authority, Milan, Italy; 11 Regional Centre for Environment and Health, Regional Environmental Protection Agency–Emilia-Romagna, Modena, Italy; 12 Epidemiology Observatory, Department of Public Health, Local Health Authority, Bologna, Italy

## Abstract

Background: Evidence on the short-term effects of fine and coarse particles on morbidity in Europe is scarce and inconsistent.

Objectives: We aimed to estimate the association between daily concentrations of fine and coarse particles with hospitalizations for cardiovascular and respiratory conditions in eight Southern European cities, within the MED-PARTICLES project.

Methods: City-specific Poisson models were fitted to estimate associations of daily concentrations of particulate matter with aerodynamic diameter ≤ 2.5 μm (PM_2.5_), ≤ 10 μm (PM_10_), and their difference (PM_2.5–10_) with daily counts of emergency hospitalizations for cardiovascular and respiratory diseases. We derived pooled estimates from random-effects meta-analysis and evaluated the robustness of results to co-pollutant exposure adjustment and model specification. Pooled concentration–response curves were estimated using a meta-smoothing approach.

Results: We found significant associations between all PM fractions and cardiovascular admissions. Increases of 10 μg/m^3^ in PM_2.5_, 6.3 μg/m^3^ in PM_2.5–10_, and 14.4 μg/m^3^ in PM_10_ (lag 0–1 days) were associated with increases in cardiovascular admissions of 0.51% (95% CI: 0.12, 0.90%), 0.46% (95% CI: 0.10, 0.82%), and 0.53% (95% CI: 0.06, 1.00%), respectively. Stronger associations were estimated for respiratory hospitalizations, ranging from 1.15% (95% CI: 0.21, 2.11%) for PM_10_ to 1.36% (95% CI: 0.23, 2.49) for PM_2.5_ (lag 0–5 days).

Conclusions: PM_2.5_ and PM_2.5–10_ were positively associated with cardiovascular and respiratory admissions in eight Mediterranean cities. Information on the short-term effects of different PM fractions on morbidity in Southern Europe will be useful to inform European policies on air quality standards.

Citation: Stafoggia M, Samoli E, Alessandrini E, Cadum E, Ostro B, Berti G, Faustini A, Jacquemin B, Linares C, Pascal M, Randi G, Ranzi A, Stivanello E, Forastiere F, the MED-PARTICLES Study Group. 2013. Short-term associations between fine and coarse particulate matter and hospitalizations in Southern Europe: results from the MED-PARTICLES project. Environ Health Perspect 121:1026–1033; http://dx.doi.org/10.1289/ehp.1206151

## Introduction

The European air quality standards are under revision, and a new directive will be delivered by the European Union (EU) in the next few years. As part of this process, the EU has indicated several specific issues of concern. Among the open issues is the extent of short-term health effects of fine and coarse particle concentrations and components in Europe, and the shape of the concentration–response relationships between fine particles and mortality and morbidity. Much of the evidence about short-term associations between fine particles [particulate matter with aerodynamic diameter ≤ 2.5 μm (PM_2.5_)] and health end points comes from studies conducted in the United States, where a 24-hr National Ambient Air Quality Standard for PM_2.5_ was first introduced in 1997 (U.S. Environmental Protection Agency 2012). From that time, data on fine particles have been collected in many parts of the United States, and evidence has accumulated suggesting significant effects of fine particles on both mortality (Ostro et al. 2006; Zanobetti and Schwartz 2009) and hospital admissions (Bell et al. 2008; Dominici et al. 2006). In contrast, EU legislation currently has a single limit value for exposure to PM_2.5_ based on an annual averaging period, without regulatory standards for daily concentrations. Only a few studies on the short-term effects of PM_2.5_ on mortality or morbidity have been conducted in Europe, with most conducted in a single city (Anderson et al. 2001; Atkinson et al. 2010; Belleudi et al. 2010; Halonen et al. 2009; Linares and Diaz 2010), and only one based on multiple cities in one country (France) (Host et al. 2008). Therefore, it is unclear whether previous findings can be generalized to all of Europe.

Another topic under debate in the EU is the role of other PM fractions on human health and, more specifically, whether coarse particles (particles with diameter 2.5–10 µm; PM_2.5–10_) are associated with health, and whether they should be monitored by European policies. This discussion was stimulated by a systematic review published by Brunekreef and Forsberg (2005), in which the authors concluded that “coarse PM has a stronger or as strong short-term effect” on respiratory health (based on emergency hospitalizations for respiratory outcomes) as fine PM. In addition, they concluded that there was some evidence supporting effects of PM_2.5–10_ on cardiovascular hospitalizations, whereas, for overall mortality, the evidence was stronger for an effect of PM_2.5_, and limited for coarse particles.

The MED-PARTICLES (“Particles size and composition in Mediterranean countries: geographical variability and short-term health effects”) project was specifically designed to address these and other related questions. It is financed by the EU under the LIFE+ framework, and aims to describe and compare the composition of airborne particles across Mediterranean cities and to estimate health effects associated with exposure to PM concentrations, PM components and sources, and Saharan dust and forest fires (MED-PARTICLES 2010).

Here we present the results of an investigation of short-term associations of daily concentrations of PM_2.5_, PM_2.5–10_, and PM_10_ (PM with aerodynamic diameter ≤ 10 μm) with emergency hospitalizations for cardiovascular and respiratory diseases in eight Southern European cities.

## Methods

*Study population.* Daily counts of emergency hospital admissions were collected from national or regional health information systems for 10 European cities, between 2001 and 2010: Milan, Turin, Bologna, Parma, Reggio Emilia, Modena, and Rome, Italy; Marseille, France; and Madrid and Barcelona, Spain. Because Parma, Reggio Emilia, and Modena are very close and share common environmental and sociodemographic characteristics, they have been analyzed altogether as a single conurbation called “Emilia Romagna.” Hospitalizations only of residents ≥ 15 years of age were considered. Two study outcomes were defined on the basis of the primary discharge diagnosis: cardiovascular hospitalizations [*International Classification of Diseases, 9th Revision* (ICD-9) codes 390–459; and *10th Revision* (ICD-10) codes I00–I99] (WHO 1999), and respiratory hospitalizations (ICD-9 codes 460–519; ICD-10 codes J00–J99). All data were extracted and collected according to a common protocol. Because data were anonymous and collected as daily counts, no informed consent was needed. And because the analysis on daily counts was conducted by a public health institute, there was no need for approval by an institutional review board.

*Environmental variables.* Daily concentrations of PM_2.5_, PM_10_, nitrogen dioxide (NO_2_), and ozone (O_3_) were collected in each city from multiple monitors belonging to air quality monitoring networks. Only monitors with complete data for ≥ 75% of the study period were accepted. When a monitor had a missing value for a specific day, it was replaced by the average of the values of the remaining stations for that day multiplied by a factor equal to the ratio of the annual mean for the missing station over the corresponding annual mean for the other stations (Katsouyanni et al. 2001). When data from all monitors were missing on a day, the daily value was left as missing. Daily mean concentrations were computed for PM and NO_2_, and daily maximum 8-hr running means were calculated for O_3_. Daily PM_2.5–10_ was calculated for each station as the difference between PM_10_ and PM_2.5_, provided that both PM fractions were measured at the same station using the same sampling methodology. Nine of 10 cities sampled PM concentrations using the gravimetric method or an equivalent one, whereas only one city, Marseille, provided uncorrected concentrations from tapered element oscillating microbalance (TEOM) samplers. Daily mean air temperature was collected from each center, using airport meteorological stations where available.

*Other confounders.* Time-varying confounders were constructed according to a common protocol, under the assumption of similar behavior of the study populations during holidays and vacation periods. They include holidays (a four-level variable assuming a value of 3 on Christmas and Easter; 2 in the periods surrounding Christmas and Easter; 1 on isolated holidays; 0 on other days); summer population decrease (a three-level variable assuming value 2 in the 2-week period around 15 August; 1 from 16 July to 31 August, except for the aforementioned period; 0 for all other days); and influenza epidemics (a two-level variable assuming 0 on normal days and 1 on days with particularly high influenza episodes). Influenza epidemics were identified using national influenza surveillance systems, where available, or were identified based on daily counts of hospitalizations for influenza (ICD-9 code 487; ICD-10 codes J09–J11).

*Statistical analysis.* The analyses were carried out using a two-stage approach. In the first stage, city-specific overdispersed Poisson regression models were fitted, in which the dependent variable was the daily count of hospitalizations, the exposure was the (lagged) daily concentration of PM_x_, and variables added for confounding adjustment. The adjustment model was defined *a priori* and, to reduce the potential for heterogeneity in the city-specific results, was the same for all cities. The confounders were long-term and seasonal time trends, air temperature, holidays, summer population decrease, and influenza epidemics. A directed acyclic graph (DAG) of the causal relationships under study is depicted in Supplemental Material, Figure S1.

Holidays, summer population decreases, and influenza epidemics were modeled using indicator variables. Time trend was adjusted for by introducing a three-way interaction term among year, month, and day of the week. This method has been demonstrated to be equivalent to a case-crossover design using a time-stratified approach to select control days (Levy et al. 2001; Lu and Zeger 2007; Maclure 1991). Two sensitivity analyses were designed to check the robustness of the results to time trend specification: *a*) a penalized spline of time trend with 50 knots per year and a smoothing parameter tuned to approximate 8 effective degrees of freedom (df) per year (Samoli et al. 2003); *b*) a penalized spline for time trend with the smoothing parameter set to minimize the absolute value of the sum of the partial autocorrelation functions (PACF) of residuals from lag 0 to 30, but requiring a minimum of 3 df per year (Katsouyanni et al. 2009). In both cases, indicator variables for days of the week were included in the models. We performed sensitivity analyses of seasonality control because a consensus on the method best suited for time trend adjustment has not been reached in the literature (Janes et al. 2005; Whitaker et al. 2007).

We controlled for the effect of temperature by modeling high and low temperatures separately. For high temperatures we calculated the average temperature on the current and previous day (lag 0–1) and fit a natural spline with 3 df on the lagged variable only for days on which the lag 0–1 temperature was higher than the median annual temperature for the city as a whole. Similarly, we adjusted for low temperatures by fitting a natural spline with 2 df for the average temperature on previous 6 days (lag 1–6) only for days on which the lag 1–6 temperature was below the median annual value for the city (Chiusolo et al. 2011). This method accounts for differences in the lag structures and effects of cold and warm temperatures on hospitalizations while reducing the correlation between the two spline terms. We also performed an analysis that adjusted for potentially prolonged effects of warm temperatures on hospitalizations by replacing the lag 0–1 temperature term from the base model with the lag 0–6 average.

Individual pollutants were added to city-specific regression models adjusted for the *a priori* covariates described above. We evaluated the lag structure of the association between PM_x_ concentrations and daily counts of cause-specific hospitalizations by applying cubic polynomial distributed lag models, with individual daily lags from lag 0 to 5 entered simultaneously in the model and constrained to follow a polynomial shape. In addition, we defined three cumulative lag structures *a priori* to represent immediate (lag 0–1), delayed (lag 2–5), and prolonged (lag 0–5) effects, and chose one of these structures as the default lag for each PM–outcome combination based on the meta-analytical results of the distributed and cumulative lag models. This choice has been applied previously (Stafoggia et al. 2010) as a compromise between *a priori* and data-driven approaches for selecting the lag structure for different exposure/outcome combinations.

The robustness of associations to adjustment for co-exposure to other pollutants was evaluated by fitting two-pollutant models with the co-pollutant modeled using the cumulative lag selected as the default for the primary exposure–outcome combination. In addition, models that included O_3_ as the co-pollutant were fit using data restricted to the warm season only (April–September), in addition to year-round data. Finally, we investigated concentration–response functions between PM_x_ and hospitalizations by fitting, for each city, a natural spline model for the exposure with two equally spaced inner knots, and pooling the city-specific estimates using a meta-smoothing approach (Schwartz and Zanobetti 2000).

In the second stage of the analysis, we pooled the city-specific results using random-effects meta-analytical procedures according to the method proposed by Jackson et al. (2010). We tested heterogeneity among the city-specific results by applying the chi-square test from Cochran’s Q statistic, and estimated the amount of heterogeneity by computing the *I*^2^ statistic (Higgins and Thompson 2002), which represents the proportion of total variation in effect estimates due to between-cities heterogeneity. We considered city-specific effect estimates to be significantly heterogeneous when *I*^2^ was > 50% and the χ^2^ *p*-value was < 0.10.

All results are expressed as percent increases in hospitalizations, with 95% CI, relative to fixed increments in each PM fraction: 10 μg/m^3^ for PM_2.5_, 6.3 μg/m^3^ for PM_2.5–10_, and 14.4 μg/m^3^ for PM_10_. These increments were chosen to represent a comparable amount of daily variability across pollutants and facilitate comparisons of effect estimates among the pollutants. More specifically, we computed the interquartile range (IQR) of each PM fraction from the joint distribution for the eight cities (six cities for PM_2.5–10_), and scaled the three resulting IQRs to express PM_2.5_ effects per 10-μg/m^3^ increase. In addition, we report selected associations in the text using a common increment of 10 μg/m^3^ for all three PM fractions.

All first-stage analyses were fit using R, version 2.15.0 (R Development Core Team; http://R-project.org). Meta-analyses were conducted using Stata, version 11 (StataCorp, College Station, TX, USA).

## Results

The population base comprised residents ≥ 15 years of age in eight Southern European cities, totaling > 11 million inhabitants (Table 1). The mean daily counts of hospitalizations ranged from 18 in the Emilia Romagna conurbation to 116 in Madrid for cardiovascular diseases, and from 8 in Bologna to 97 in Madrid for respiratory conditions. The study periods were recent and comparable across cities, except for Marseille with 2001–2003 data.

**Table 1:** Study populations and emergency hospital admissions for cardiovascular and respiratory causes among residents ≥ 15 years of age in the eight cities of the MED-PARTICLES project.

City^*a*^	Study period	Population		Cardiovascular admissions			Respiratory admissions		
		Date	*n*	*n*	*n *per 1,000 person-years	Daily mean	*n*	*n *per 1,000 person-years	Daily mean
Milan	2006–2010	1 January 2008	1,299,633	71,779	11.0	39.3	34,427	5.3	18.9
Turin	2006–2010	1 January 2008	908,263	48,967	10.8	26.8	21,761	4.8	11.9
Emilia Romagna^*b*^	2008–2010	1 January 2009	529,699	19,717	12.4	18.0	10,164	6.4	9.3
Bologna	2006–2010	1 January 2008	372,256	34,568	18.6	18.9	14,103	7.6	7.7
Marseille	2001–2003	Census 1999	796,525	46,905	19.6	42.8	18,069	7.6	16.5
Rome	2006–2010	1 January 2008	2,718,768	153,176	11.3	83.9	53,825	4.0	29.5
Barcelona	2003–2010	1 January 2007	1,595,110	151,426	11.9	51.8	139,062	10.9	47.6
Madrid	2004–2009	1 January 2007	3,132,503	201,041	13.5	115.9	167,850	11.3	96.7
Total	—	—	11,352,757	727,579	12.6	51.4	459,261	7.9	32.5
^***a***^Cities are ordered by latitude, north to south. ^***b***^Emilia Romagna includes Parma, Reggio Emilia, and Modena.									

Pollutant concentrations and air temperature data for each city are summarized in Supplemental Material, Tables S1 (year-round) and S2 (separate PM concentration distributions for the cold and warm seasons). Daily concentrations of PM_10_ and PM_2.5_ were highest in Milan and Turin. Madrid had the smallest daily PM_2.5_ concentrations and the largest PM_2.5–10_ concentrations, resulting in a fine:coarse particles ratio that was much smaller for this city (< 1) than the others (ranging from 1.5 to 2.2). NO_2_ and O_3_ concentrations were similar among the cities, whereas temperature displayed a mild increasing north–south gradient. Pearson correlation coefficients between PM_2.5_ and PM_2.5–10_ were close to 0 in Barcelona, Marseille, and Rome (the cities closest to the sea and therefore likely to be more affected by sea winds), but were ≥ 0.5 in the other cities (data not shown). NO_2_ concentrations were highly correlated with both PM_2.5_ and PM_10_ (> 0.6 for all cities except Barcelona), whereas correlations between NO_2_ and PM_2.5–10_ ranged from 0.17 in Marseille to 0.57 in Madrid.

Pooled estimates from polynomial distributed lag models (Supplemental Material, Figure S2) clearly show evidence of an immediate effect of the three PM fractions on cardiovascular hospitalizations, up to lag 1, whereas associations with respiratory admissions were evident until day 5.

Table 2 reports associations between PM and hospitalizations for the three cumulative lags (0–1, 2–5, and 0–5 days). Significant positive associations of a comparable magnitude were estimated for all PM fractions with cardiovascular hospitalizations: increments of 10 μg/m^3^ in PM_2.5_, 6.3 μg/m^3^ in PM_2.5–10_, and 14.4 μg/m^3^ in PM_10_ (lag 0–1) were associated with 0.51% (95% CI: 0.12, 0.90%), 0.46% (95% CI: 0.10, 0.82%), and 0.53% (95% CI: 0.06, 1.00%) increases in cardiovascular admissions, respectively. When expressed per 10 μg/m^3^, corresponding estimates for PM_2.5–10_ and PM_10_ were 0.73% (95% CI: 0.16, 1.30%) and 0.36% (95% CI: 0.04, 0.69%), suggesting an effect of coarse particles around 40% higher than that of PM_2.5_ for the same increment. Associations between cardiovascular hospitalizations and PM were null for lag 2–5, and smaller or null for lag 0–5 (Table 2). Given these results and the results of the distributed lag models, we used lag 0–1 for subsequent analyses of cardiovascular hospitalizations.

**Table 2:** Associations between PM and hospitalizations per IQR increases of 10, 6.3, and 14.4 μg/m^3^ for PM_2.5_, PM_2.5–10_ and PM_10_, respectively.

Pollutant	Lag	Cardiovascular admissions			Respiratory admissions		
		Percent increase (95% CI)	χ^2^ *p*-value	*I*^2^	Percent increase (95% CI)	χ^2^ *p*-value	*I*^2^
PM_2.5_
	0–1	0.51 (0.12, 0.90)	0.20	29	0.49 (–0.12, 1.09)	0.16	34
	2–5	0.15 (–0.22, 0.53)	0.82	0	1.07 (0.04, 2.11)	0.00	71
	0–5	0.49 (0.03, 0.95)	0.70	0	1.36 (0.23, 2.49)	0.01	65
PM_2.5–10_
	0–1	0.46 (0.10, 0.82)	0.33	13	0.60 (0.08, 1.13)	0.26	23
	2–5	–0.38 (–0.89, 0.12)	0.23	28	0.75 (–0.71, 2.23)	0.00	77
	0–5	0.05 (–0.68, 0.78)	0.09	47	1.24 (–0.32, 2.82)	0.00	74
PM_10_
	0–1	0.53 (0.06, 1.00)	0.06	48	0.65 (0.20, 1.10)	0.38	6
	2–5	–0.06 (–0.43, 0.31)	0.47	0	0.95 (–0.03, 1.94)	0.00	68
	0–5	0.30 (–0.14, 0.74)	0.49	0	1.15 (0.21, 2.11)	0.04	52

Associations with respiratory hospitalizations were strongest for all three PM fractions at the cumulative lag 0–5, though the association was not statistically significant for PM_2.5–10_, and effect estimates were highly heterogeneous across cities. Specifically, lag 0–5 effect estimates were 1.36% (95% CI: 0.23, 2.49%) for a 10-μg/m^3^ increase in PM_2.5_, 1.24% (95% CI: –0.32, 2.82%) for a 6.3-μg/m^3^ increase in PM_2.5–10_, and 1.15% (95% CI: 0.21, 2.11%) for a 14.4-μg/m^3^ increase in PM_10_. Associations were more homogeneous among cities for lag 0–1, and statistically significant for the coarse fraction (0.60%; 95% CI: 0.08, 1.13%) and PM_10_ (0.65%; 95% CI: 0.20, 1.10%). When expressed for increments of 10 μg/m^3^, the association with respiratory admissions was strongest for coarse particles (1.95%; 95% CI: –0.51, 4.48% for lag 0–5). We used lag 0–5 for subsequent analyses of association between the PM fractions and respiratory hospitalizations.

Pooled estimates from one- and two-pollutant models are reported in Table 3. When evaluated simultaneously, the association between fine particles and cardiovascular hospitalizations (lag 0–1) was unaffected by adjustment for PM_2.5–10_ (0.49% increase per 10 μg/m^3^ PM_2.5_; 95% CI: 0.06, 0.91%) but adjusting for PM_2.5_ decreased the association between cardiovascular hospitalizations and coarse PM (0.28% increase per 6.3 μg/m^3^ PM_2.5–10_; 95% CI: –0.09, 0.66%). Associations of fine and coarse particles with respiratory admissions (lag 0–5) both decreased considerably with mutual adjustment (to 0.55%; 95% CI: –0.29, 1.40 for PM_2.5_, and 0.66%; 95% CI: –0.78, 2.13 for PM_2.5–10_). Adjustment for NO_2_ decreased association between PM_2.5–10_ and both outcomes, whereas it decreased the association between PM_2.5_ and cardiovascular hospitalizations, but strengthened the association with respiratory admissions. However, given the high correlation between PM_2.5_ and NO_2_ in all cities, results from two-pollutant models must be interpreted with caution. No confounding from O_3_ was apparent in the all-year analysis, nor in the analysis restricted to the warm period. However, associations of PM_2.5_ and PM_2.5–10_ with both outcomes were much stronger and always statistically significant when the analyses were restricted to the warm period (April–September) both with and without adjustment for O_3_.

**Table 3:** Associations between PM and hospitalizations from one- and two-pollutant models, per increases of 10, 6.3, and 14.4 μg/m^3^ for PM_2.5_, PM_2.5–10_, and PM_10_, respectively.

Pollutant	Period	Cardiovascular admissions (lag 0–1)			Respiratory admissions (lag 0–5)		
		Percent increase (95% CI)	χ^2^ *p*-value	*I*^2^	Percent increase (95% CI)	χ^2^ *p*-value	*I*^2^

PM_2.5_	All year	0.51 (0.12, 0.90)	0.20	29	1.36 (0.23, 2.49)	0.01	65
+ PM_2.5–10_	All year	0.49 (0.06, 0.91)	0.48	0	0.55 (–0.29, 1.40)	0.47	0
+ NO_2_	All year	0.21 (–0.28, 0.69)	0.21	28	1.63 (0.30, 2.97)	0.05	51
+ O_3_	All year	0.49 (0.06, 0.91)	0.18	31	1.13 (0.05, 2.23)	0.04	53
PM_2.5_	April–September	1.76 (0.68, 2.84)	0.18	32	4.49 (1.72, 7.35)	0.04	51
+ O_3_	April–September	2.11 (1.13, 3.10)	0.34	11	4.51 (1.35, 7.77)	0.03	56

PM_2.5–10_	All year	0.46 (0.10, 0.82)	0.33	13	1.24 (–0.32, 2.82)	0.00	74
+ PM_2.5_	All year	0.28 (–0.09, 0.66)	0.85	0	0.66 (–0.78, 2.13)	0.07	51
+ NO_2_	All-year	0.25 (–0.13, 0.62)	0.42	0	0.99 (–0.71, 2.73)	0.02	61
+ O_3_	All year	0.48 (0.14, 0.83)	0.41	2	1.17 (–0.45, 2.81)	0.01	70
PM_2.5–10_	April–September	0.90 (0.28, 1.52)	0.33	13	3.01 (0.68, 5.39)	0.07	51
+ O_3_	April–September	0.93 (0.37, 1.49)	0.50	0	3.03 (0.44, 5.68)	0.05	54

PM_10_	All year	0.53 (0.06, 1.00)	0.06	48	1.15 (0.21, 2.11)	0.04	52
+ NO_2_	All year	0.19 (–0.42, 0.79)	0.05	50	1.33 (–0.02, 2.69)	0.07	47
+ O_3_	All year	0.50 (–0.03, 1.03)	0.04	52	0.99 (0.13, 1.86)	0.17	32
PM_10_	April–September	2.07 (1.30, 2.85)	0.61	0	4.33 (2.59, 6.11)	0.32	14
+ O_3_	April–September	2.25 (1.45, 3.06)	0.83	0	4.98 (1.98, 8.06)	0.07	47

The robustness of the main findings was checked against model specification in three sensitivity analyses, whose results are summarized in Table 4. In general, the case-crossover method adopted in the base model for the adjustment of time trend provided effect estimates quite consistent with the spline method with minimization of the PACF of residuals, whereas associations based on models that used the spline method with 8 df per year were weaker for all particle metrics and both study outcomes. In addition, associations between all three PM fractions and respiratory admissions were strongest when estimated using the spline method with the PACF criterion. Effect estimates from the sensitivity model adjusted for prolonged effects of warm temperatures were consistent with base model estimates.

**Table 4:** Association between PM and hospitalizations for different model specifications, per increases of 10, 6.3, and 14.4 μg/m^3^ for PM_2.5_, PM_2.5–10_, and PM_10_, respectively.

Pollutant/model	Cardiovascular admissions (lag 0–1)			Respiratory admissions (lag 0–5)		
	Percent increase (95% CI)	χ^2^ *p*-value	*I*^2^	Percent increase (95% CI)	χ^2^ *p*-value	*I*^2^
PM_2.5_
Base model	0.51 (0.12, 0.90)	0.20	29	1.36 (0.23, 2.49)	0.01	65
Trend, 8 df/year	0.28 (–0.12, 0.68)	0.11	41	0.87 (0.03, 1.72)	0.08	46
Trend, minimum PACF^*a*^	0.47 (0.14, 0.80)	0.23	25	1.64 (0.63, 2.65)	0.00	66
Temperature, lag 0–6 for warm period	0.52 (0.13, 0.91)	0.17	32	1.48 (0.29, 2.69)	0.00	69
PM_2.5–10_
Base model	0.46 (0.10, 0.82)	0.33	13	1.24 (–0.32, 2.82)	0.00	74
Trend, 8 df/year	0.34 (0.05, 0.63)	0.91	0	1.10 (0.01, 2.20)	0.05	55
Trend, minimum PACF^*a*^	0.53 (0.25, 0.81)	1.00	0	1.69 (0.26, 3.13)	0.00	72
Temperature, lag 0–6 for warm period	0.57 (0.26, 0.89)	0.50	0	1.40 (–0.27, 3.10)	0.00	76
PM_10_
Base model	0.53 (0.06, 1.00)	0.06	48	1.15 (0.21, 2.11)	0.04	52
Trend, 8 df/year	0.31 (–0.05, 0.67)	0.18	31	0.96 (0.31, 1.61)	0.26	22
Trend, minimum PACF^*a*^	0.56 (0.28, 0.84)	0.40	4	1.72 (0.85, 2.59)	0.03	55
Temperature, lag 0–6 for warm period	0.57 (0.12, 1.02)	0.07	47	1.29 (0.28, 2.32)	0.02	58
^***a***^Estimated degrees of freedom per year were 3 (Milan, Turin, Emilia-Romagna, Bologna, Barcelona), 4(Rome), and 5(Marseille, Madrid) for cardiovascular hospitalization; and 3 (Turin, Bologna), 4(Marseille, Madrid), 5 (Rome), 6 (Milan, Emilia-Romagna), and 9 (Barcelona) for respiratory hospitalizations.						

**Figure 1:**
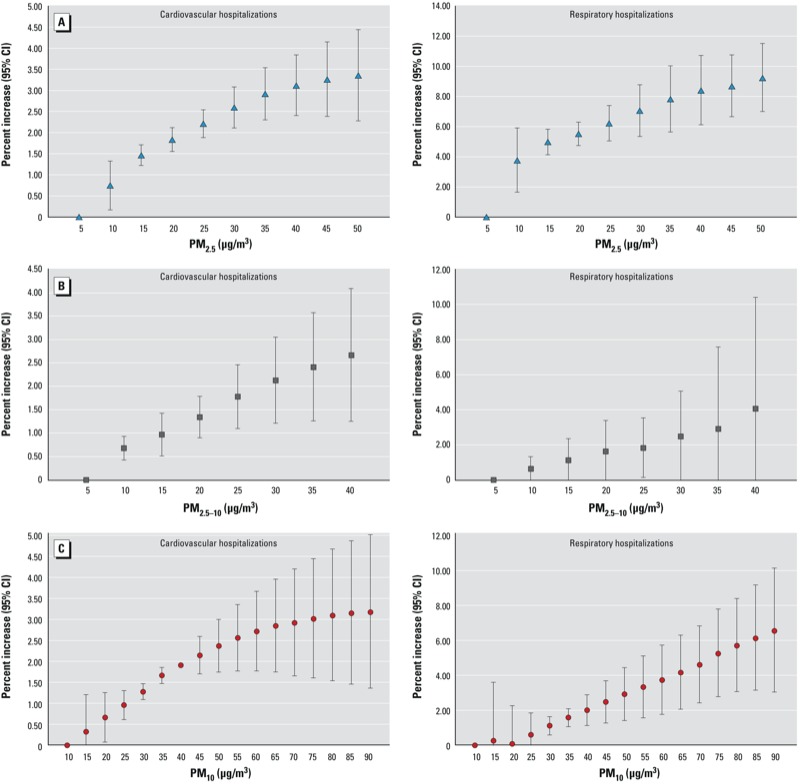
Concentration–response relationship between PM_2.5_ (*A*), PM_2.5–10_ (*B*), and PM_10_ (*C*) with cardiovascular hospitalizations, lag 0–1 (left), and respiratory hospitalizations, lag 0–5 (right). Values are percent increase (95% CI) of hospital admissions associated with increases of PM_2.5_ and PM_2.5–10_ levels relative to 5 μg/m^3^, and of PM_10_ levels relative to 10 μg/m^3^.

Finally, Figure 1 reports the concentration–response relationships between PM_2.5_, PM_2.5–10_, and PM_10_ with cardiovascular hospitalizations (lag 0–1) and respiratory hospitalizations (lag 0–5). All estimates are reported as percentage increases in hospital admissions (95% CI) associated with increasing concentrations of PM_2.5_ and PM_2.5–10_ relative to 5 μg/m^3^, and of PM_10_ relative to 10 μg/m^3^. Associations of PM_2.5_ and PM_10_ with cardiovascular hospitalizations seemed to be consistent with two linear functions, with a steeper slope for lower concentrations. There was no visual evidence of a departure from linearity for PM_2.5–10_ and cardiovascular hospitalizations, or for any of the PM metrics and respiratory admissions.

## Discussion

We investigated the association between daily concentrations of fine and coarse particles and hospitalizations for cardiorespiratory conditions in eight Mediterranean cities, finding evidence of harmful effects of the PM metrics on both study outcomes.

Evidence of short-term effects of particulate matter on mortality and morbidity has been described widely in the literature. Although the first multicenter studies focused on PM_10_ as the main exposure (Katsouyanni et al. 2001; Samet et al. 2000), more recently interest has shifted to specific PM fractions, constituents, and sources, with the aim of better elucidating the underlying biological mechanisms. It has been argued that the fine fraction of PM is the one more responsible for the health effects, because it includes toxic components such as nitrates, sulfates, acids, and metals originating from combustion processes, and it can deposit more deeply into the lungs (Pope and Dockery 2006). In contrast, coarse particles are dominated by crustal materials, resuspended dust, sea salts, desert dust, and biogenic components including pollen, spores, and other plant parts (Pope and Dockery 2006). A systematic review conducted by Brunekreef and Forsberg (2005) pointed toward a need to further investigate the health effects of PM_2.5–10_ based on evidence that coarse and fine PM exert similar effects on respiratory outcomes, and on evidence supporting an association between PM_2.5–10_ and cardiovascular outcomes.

Recent multicenter studies focusing on the association between PM_2.5_ and hospitalizations in adult populations have been mainly conducted in North America (Bell et al. 2008; Dominici et al. 2006; Stieb et al. 2009; Zanobetti et al. 2009). Dominici et al. (2006) investigated the effects of PM_2.5_ on cardiovascular and respiratory hospital admissions in 204 U.S. urban counties within the National Morbidity, Mortality, and Air Pollution Study (NMMAPS). They estimated statistically significant increases in hospitalizations associated with a 10-μg/m^3^ increase in PM_2.5_ for a number of specific cardiovascular conditions, ranging from 0.44% (95% CI: 0.02, 0.86%) for ischemic heart disease hospitalizations to 1.28% (95% CI: 0.78, 1.78%) for heart failure admissions. Positive associations were also reported for hospital admissions for specific respiratory outcomes (0.91%; 95% CI: 0.18, 1.64% for PM_2.5_ on the same day and chronic obstructive pulmonary disease, and 0.92%; 95% CI: 0.41, 1.43% for respiratory tract infection, with a 2-day lag). Similar results for cardiovascular hospitalizations were reported for another NMMAPS study (Bell et al. 2008) of 202 U.S. counties, which estimated a 0.8% increase in cardiovascular admissions (95% posterior interval: 0.59, 1.01) per 10-μg/m^3^ increase in lag 0 PM_2.5_. The same study reported a 0.41% increase (95% posterior interval: 0.09, 0.74) in all respiratory admissions associated with 10-μg/m^3^ increase in lag 2 PM_2.5_, a weaker association than reported by Dominici et al. (2006) for specific respiratory conditions. Zanobetti et al. (2009) investigated associations of PM_2.5_ concentrations and components with cause-specific admissions in 26 U.S. communities, and estimated stronger associations with cardiac and respiratory diseases than previous studies: specifically, a 1.89% increase (95% CI: 1.34, 2.45) in cardiac admissions and a 2.07% increase (95% CI: 1.20, 2.95) in respiratory admissions in association with 10-μg/m^3^ increase in lag 0–1 PM_2.5_. The authors concluded that “particles originating from industrial combustion processes or traffic may, on average, have greater toxicity.” However, evidence from European studies has been weaker. One six-city study conducted in France (Host et al. 2008) reported large and statistically significant associations between PM_2.5_ and cardiovascular admissions (0.9% increase; 95% CI: 0.1, 1.8 with a 10-μg/m^3^ increase in lag 0–1 PM_2.5_), but no associations between PM_2.5_ and respiratory diseases. Results from single-city European investigations (Anderson et al. 2001; Atkinson et al. 2010; Belleudi et al. 2010; Halonen et al. 2009; Linares and Diaz 2010) have been inconsistent.

Epidemiological studies on the short-term effects of PM_2.5–10_ on hospitalizations are few and inconsistent. In a large U.S. study of 108 counties (Peng et al. 2008), a 10-μg/m^3^ increase in PM_2.5–10_ was associated with a 0.36% (95% posterior interval: 0.05, 0.68) increase in cardiovascular disease admissions on the same day. However, when adjusted for PM_2.5_, the association was still positive but no longer statistically significant. Two studies conducted in the United Kingdom (Anderson et al. 2001; Atkinson et al. 2010) did not identify any evidence of effects of coarse particles on hospital admissions for either cardiovascular or respiratory causes. A study conducted in six French cities (Host et al. 2008) found no association between coarse PM and cardiovascular or respiratory admissions, and reported only one statistically significant association with ischemic heart disease in the elderly. We identified positive associations of coarse particles with both cardiovascular (0.46% increase; 95% CI: 0.10, 0.82 with a 6.3-μg/m^3^ increase in lag 0–1 PM_2.5–10_) and respiratory admissions (1.24% increase; 95% CI: –0.32, 2.82) with a 6.3-μg/m^3^ increase in lag 0–5 PM_2.5–10_). Corresponding associations with a 10-μg/m^3^ increase in PM_2.5–10_ were higher than previously reported (0.73%; 95% CI: 0.16, 1.30%, and 1.95%; 95% CI: –0.51, 4.48%, respectively). However, because PM_2.5–10_ is obtained as the difference between PM_10_ and PM_2.5,_ it is affected by measurement error from two sources, and the direction and magnitude of the resulting bias in the associations with hospitalizations cannot be predicted.

Our study is, to our knowledge, the first study involving cities from multiple countries in Southern Europe and investigating the health effects of fine and coarse particles on cause-specific hospitalizations. Our estimates of associations between PM_2.5_ and cardiovascular admissions are slightly smaller than those reported by U.S. studies, whereas associations between PM_2.5_ and respiratory admissions, and associations between both outcomes and PM_2.5–10_, are more similar. Several differences must be acknowledged between our study and the North American ones. First, the chemical mixture of PM_2.5_ in Europe is likely to be different from that in the United States. For example, diesel powered cars make up 50% of the fleet in Europe and only about 2% in the United States. Consistent with this, elemental carbon concentrations estimated for 187 counties in the United States were about 0.6 μg/m^3^ (Bell 2012) compared with about 1.5 μg/m^3^ for Madrid and Barcelona (unpublished data). Second, PM concentrations for most U.S. studies were measured every 3rd or 6th day, whereas daily PM measurements were available for the eight cities in the present study. In addition, our study, along with the entire MED-PARTICLES project, comprises Southern European cities characterized by highly urbanized areas with intense traffic congestion, elevated sea traffic due to tourist and shipping activities over the Mediterranean area, mild meteorological conditions favoring outdoor activities during most of the year, enhanced formation of secondary pollutants owing to intense solar radiation, high frequency of wildfires, and Saharan dust advection episodes, especially in summer and spring.

Associations between fine particles and cardiovascular hospitalizations in our study were not affected by coarse PM co-exposure, but the associations of both fine and coarse PM with respiratory admissions decreased to nonsignificance when evaluated together in two-pollutant models. Because of moderate to high correlations between the two exposures, it is difficult to disentangle their potential effects. This problem was even more apparent in two-pollutant models involving PM_2.5_ and NO_2_. The respiratory effects were highly heterogeneous, and there are no simple explanations for that. These heterogeneous effects were already seen in a large Italian study (Faustini et al. 2013). City-specific prevalence of patients with chronic respiratory conditions may be different, as may their individual level of response to air pollutants on different seasons.

An additional contribution of the present study is the strategy for time-trend adjustment. We applied three different approaches to adjust for the confounding effect of long-term and seasonal time trends: a case-crossover approach that used three-way interaction terms among year, month, and day of the week (84 df per year) to adjust for time trends; a penalized spline and a smoothing parameter with 8 df per year; and a penalized spline with df per year selected to minimize PACF residuals (3–5 df per year for cardiovascular admissions, and 3–9 df per year for respiratory admissions). Despite the variation in the degrees of control, the three methods provided consistent results, suggesting little residual confounding due to long-term and seasonal time trends.

We estimated much stronger associations of fine and coarse particles with hospitalizations for both cardiovascular and respiratory conditions during the warm period compared with the cold period. This is consistent with the worldwide literature (Faustini et al. 2013; Stieb et al. 2009; Strickland et al. 2010). Two complementary hypotheses have been suggested: First, exposures to air pollutants may increase during warm months because of increased outdoor activities and open windows (Stafoggia et al. 2008); second, the air pollution mixture may include a greater proportion of toxic components during warmer months (Peng et al. 2005). Both explanations are plausible during the warm season in the Mediterranean area because of seasonal differences in PM composition (Perrino et al. 2009; Querol et al. 2009; van Drooge et al. 2012) and an increased frequency of wildfires and Saharan dust episodes, whose particles have been related to health effects (Hänninen et al. 2009; Karanasiou et al. 2012). These aspects have been already documented in the Southern Mediterranean countries (Querol et al. 2009), and further results will be available from the MED-PARTICLES project.

To our knowledge, this is the first study to investigate the concentration–response functions of fine and coarse particles with cardiovascular and respiratory morbidity. Epidemiological studies of the short-term effects of PM_10_ on cause-specific mortality (Daniels et al. 2004; Faustini et al. 2011; Katsouyanni et al. 2009) have reported no apparent departures from linearity. An extension of the Harvard Six Cities Study also reported no evidence of a departure from linearity for the relationship between long-term exposure to PM_2.5_ and survival (Schwartz et al. 2008). In the present study we found a suggestion of linear effects of all PM metrics on respiratory hospitalizations, and of PM_2.5–10_ on cardiovascular admissions. However, the slopes of estimated exposure–response curves for cardiovascular admissions in association with PM_10_ and PM_2.5_ were steeper for low to moderate concentrations (approximately 30 μg/m^3^ for PM_2.5_ and 60 μg/m^3^ for PM_10_) than higher concentrations, consistent with the dose–response curve previously estimated for the long-term effects of PM_2.5_ on mortality from ischemic heart disease (Pope et al. 2009). These results need further investigation in other locations and with other analytical approaches, but they support effects of all PM metrics on cardiovascular and respiratory conditions at low concentrations, including concentrations below the current EU air quality standards.

We believe that our results have clear implications for the EU policy on air quality. Specifically, they suggest that the current 24-hr daily limit value for PM_10_ in Europe is not sufficient to protect the population from short-term effects, and that a daily limit value for PM_2.5_ is clearly warranted. Both PM_2.5_ and PM_10_ daily limits are needed to control air pollution generated from different sources: vehicle exhausts and combustion sources for fine particles, natural sources together with resuspension of road dust (containing a mixture of soil, tire wear, and brake wear), non-exhaust emissions, and commercial and industrial residues for coarse particles. Because coarse particles of natural origin cannot be controlled, policy measures aimed at controlling anthropogenic sources of coarse particles would be advisable. Alternatively, a specific short-term limit value for coarse particles (PM_2.5_–PM_10_) may be considered, but only in addition to an effective PM_2.5_ daily limit.

## Conclusions

In summary, we estimated significant short-term effects of fine and coarse particles on cardiovascular and respiratory hospitalizations in eight Southern European cities. Associations were similar across PM metrics, stronger but more heterogeneous for respiratory admissions, much more pronounced during the warm period, and robust to time-trend specification. In addition, we did not find strong evidence of departure from linearity in the range of pollutant concentrations measured for the associations under investigation. These findings will help inform planned revisions of EU air quality standards and support legislation on daily PM_2.5_ concentrations to better target policies on air pollution in Europe.

## Supplemental Material

(627 KB) PDF
