# Supplementary material for: Short-term Associations between Fine and Coarse Particulate Matter and Hospitalizations in Southern Europe: Results from the MED-PARTICLES Project
Source: Environ Health Perspect. 2013 Jun 18;121(9):1026–33. doi: 10.1289/ehp.1206151 (PMC3764077; doi:10.1289/ehp.1206151)
Supplement: (627 KB) PDF [file ehp.1206151.s001.pdf]

Supplemental Material

Short-term Associations between Fine and Coarse Particulate Matter and Hospitalizations in Southern Europe: Results from the MED-PARTICLES Project

Massimo Stafoggia, Evangelia Samoli, Ester Alessandrini, Ennio Cadum, Bart Ostro, Giovanna Berti, Annunziata Faustini, Benedicte Jacquemin, Cristina Linares, Mathilde Pascal, Giorgia Randi, Andrea Ranzi, Elisa Stivanello, and Francesco Forastiere; the MED-PARTICLES Study Group.

Table of Contents

Table S1 Page 2

Table S2 Page 3

Figure S1 Page 4

Figure S2 Page 5

Table S1. Environmental variables. Daily values of particulate matter concentrations, gases and air temperature in the 8 cities of MED-PARTICLES

		$PM_{2.5}$ (μg/m[3])		$PM_{2.5\text{-}10}$ (μg/m[3])		PM_{10} (μg/m[3])		NO_2 (μg/m[3])	O_3 (April-September) (μg/m[3])	Air temperature (°C)
**City[a]**	**Study period**	**Days (*n*)**	**mean ± SD (IQR)**	**Days (*n*)**	**mean ± SD (IQR)**	**Days (*n*)**	**mean ± SD (IQR)**	**mean ± SD**	**mean ± SD**	**mean ± SD**
**Milan**	2006-2010	1,638	32.9 ± 27.1 (31.7)	1,637	14.8 ± 11.5 (11.9)	1,825	46.9 ± 34.3 (36.6)	60.0 ± 23.8	94.5 ± 31.7	13.9 ± 8.3
**Turin**	2006-2010	1,692	34.4 ± 28.3 (34.0)	-	-	1,622	48.1 ± 36.4 (44.0)	59.8 ± 25.2	106.9 ± 30.9	12.7 ± 8.0
**Emilia Romagna**	2008-2010	1,093	21.6 ± 14.9 (15.3)	1,093	12.6 ± 6.7 (7.7)	1,096	34.3 ± 19.3 (21.3)	43.3 ± 15.8	104.1 ± 28.7	14.6 ± 8.6
**Bologna**	2006-2010	1,767	25.8 ± 18.5 (18.0)	-	-	1,729	38.4 ± 21.6 (24.0)	50.1 ± 18.2	93.2 ± 31.5	14.7 ± 8.6
**Marseille**	2001-2003	1,085	18.6 ± 8.0 (11.2)	840	9.3 ± 4.3 (6.0)	1,060	26.7 ± 10.0 (14.0)	49.9 ± 15.3	108.2 ± 25.7	15.8 ± 7.1
**Rome**	2006-2010	1,818	19.6 ± 10.0 (11.1)	1,816	13.2 ± 6.9 (8.2)	1,824	34.5 ± 14.1 (15.9)	59.2 ± 16.9	96.4 ± 21.7	15.9 ± 7.0
**Barcelona**	2003-2010	2,676	23.7 ± 11.5 (12.9)	2,669	12.8 ± 10.4 (12.9)	2,669	36.5 ± 17.2 (20.4)	40.1 ± 17.3	79.7 ± 20.5	14.3 ± 6.3
**Madrid**	2004-2009	1,685	17.2 ± 9.7 (11.2)	1,681	17.5 ± 11.9 (13.0)	1,716	35.1 ± 20.1 (23.7)	62.7 ± 26.6	56.4 ± 15.1[b]	15.1 ± 7.7

[a] Cities are ordered by latitude, North to South

[b] Daily mean instead of daily maximum 8-hr running mean

Table S2. Environmental variables, by cold and warm season. Daily values of particulate matter concentrations in the 8 cities of MED-PARTICLES. The cold season is defined as October-March, the warm season is defined as April-September

	$PM_{2.5}$ (μg/m³)		$PM_{2.5-10}$ (μg/m³)		PM_{10} (μg/m³)	
**City/Season**	**Days (*n*)**	**mean ± SD (IQR)**	**Days (*n*)**	**mean ± SD (IQR)**	**Days (*n*)**	**mean ± SD (IQR)**
**Cold season (October-March)**						
**Milan**	836	48.3 ± 29.3 (40.0)	835	17.5 ± 13.5 (15.4)	910	65.4 ± 38.6 (49.1)
**Turin**	858	51.3 ± 30.3 (43.0)	-	-	838	68.0 ± 39.8 (54.0)
**Emilia Romagna**	544	29.6 ± 16.9 (22.2)	544	14.1 ± 7.9 (10.6)	547	44.0 ± 21.8 (27.7)
**Bologna**	887	34.8 ± 21.5 (24.9)	-	-	868	49.1 ± 24.4 (29.0)
**Marseille**	538	19.5 ± 9.0 (13.0)	446	8.4 ± 3.7 (5.0)	530	26.8 ± 10.9 (14.0)
**Rome**	904	22.9 ± 11.9 (15.5)	903	12.8 ± 7.2 (9.5)	910	37.7 ± 16.4 (22.3)
**Barcelona**	1,287	25.7 ± 13.8 (16.0)	1,280	11.7 ± 10.4 (10.7)	1,280	37.3 ± 19.4 (22.7)
**Madrid**	872	17.6 ± 10.3 (12.2)	872	18.6 ± 13.8 (16.3)	904	36.9 ± 22.8 (28.1)
**Warm season (April-September)**						
**Milan**	802	16.8 ± 9.7 (10.2)	802	12.0 ± 7.9 (10.4)	915	28.4 ± 13.2 (15.9)
**Turin**	834	17.0 ± 9.3 (12.0)	-	-	784	26.9 ± 13.5 (15.0)
**Emilia Romagna**	549	13.5 ± 5.6 (6.7)	549	11.1 ± 4.8 (5.3)	549	24.7 ± 9.0 (11.3)
**Bologna**	880	16.8 ± 7.8 (8.0)	-	-	861	27.6 ± 10.2 (11.0)
**Marseille**	547	17.7 ± 6.7 (10.1)	394	10.3 ± 4.7 (5.0)	530	26.6 ± 9.1 (13.0)
**Rome**	914	16.3 ± 6.0 (7.8)	913	13.6 ± 6.5 (7.0)	914	31.4 ± 10.4 (11.7)
**Barcelona**	1,389	22.0 ± 8.6 (10.5)	1,389	13.9 ± 10.3 (11.4)	1,389	35.8 ± 14.9 (18.9)
**Madrid**	813	16.8 ± 9.0 (10.2)	809	16.4 ± 9.3 (10.3)	812	33.1 ± 16.2 (18.8)

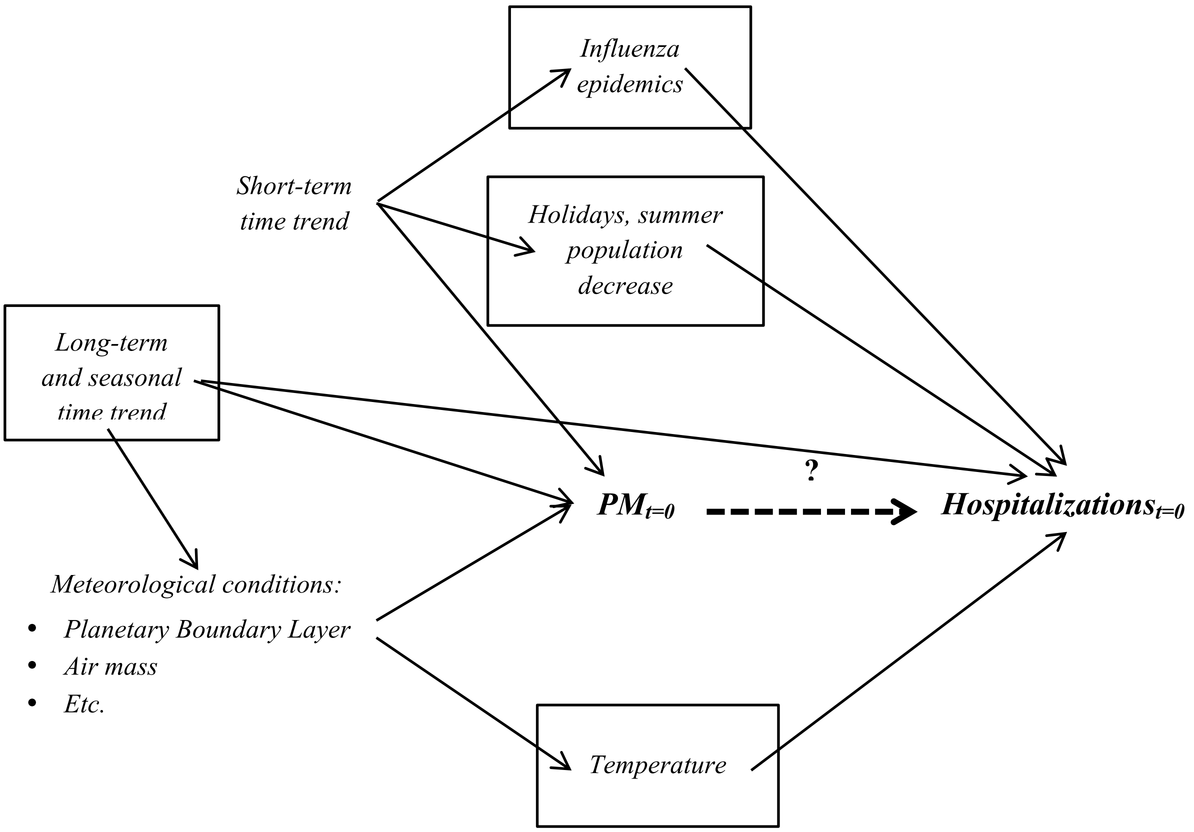

Figure S1. Directed acyclic graph (DAG) of the causal relationships between PM exposure, hospitalizations, and confounders. All variables are assumed at lag 0.

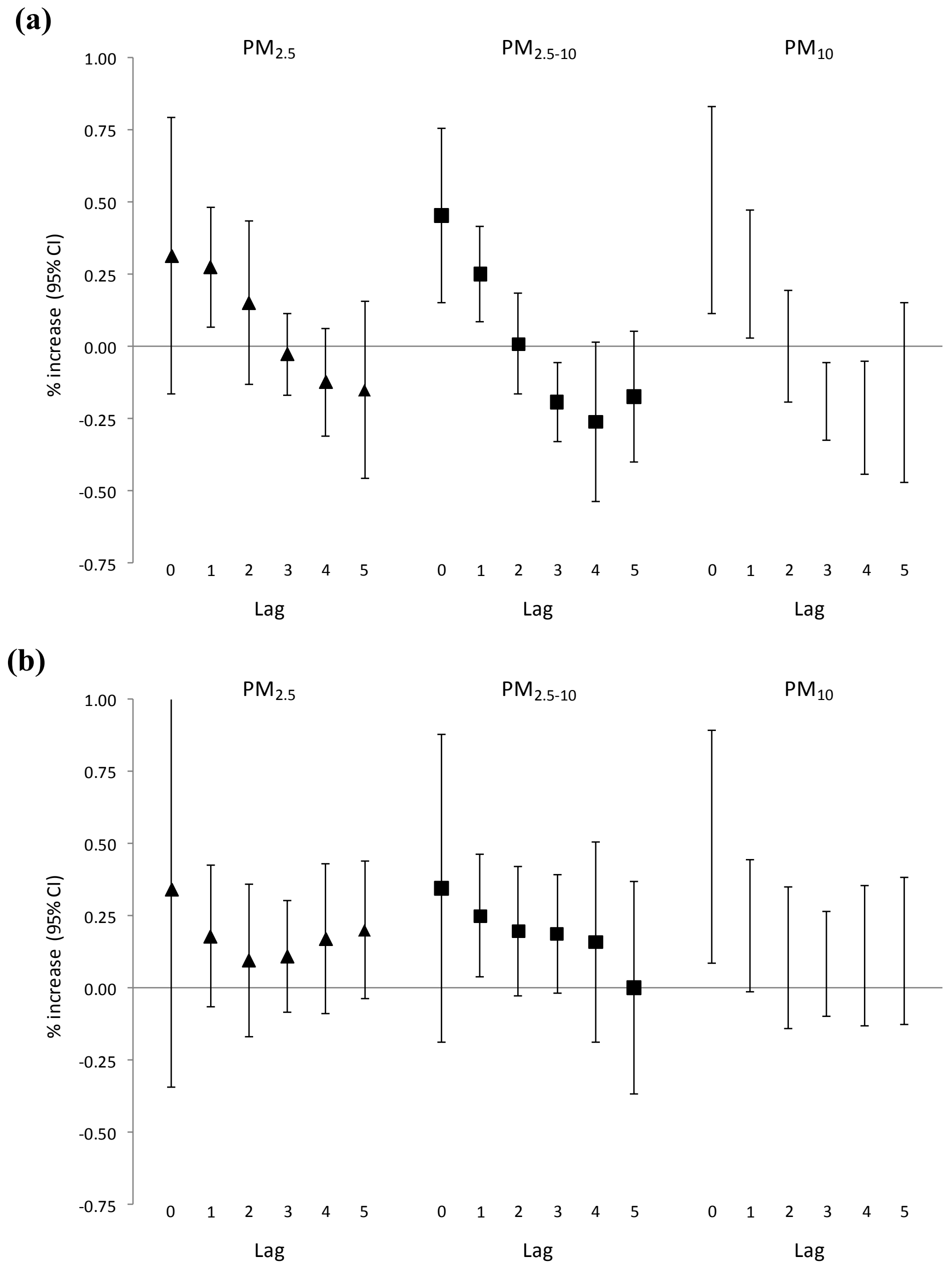

Figure S2. Association between PM and cardiovascular hospitalizations (a) and respiratory hospitalizations (b) from polynomial distributed lag models, lag 0 to 5: percentage increase of hospital admissions (95% CI) associated with increases of 10, 6.3 and 14.4 μg/m^3 for $PM_{2.5}$, $PM_{2.5-10}$ and PM_{10}, respectively. Models adjusted for time trend, high temperatures (lag 0-1), low temperatures (lag 1-6), holidays, summer population decrease, day of the week, and influenza epidemics.
